# Self-assembly and label-free fluorescent aptasensor based on deoxyribonucleic acid intercalated dyes for detecting lactoferrin in milk powder

**DOI:** 10.3389/fnut.2022.992188

**Published:** 2022-09-15

**Authors:** Jiahui Liu, Tengfei Li, Hongwei Qin, Linsen Li, Mengmeng Yan, Chao Zhu, Feng Qu, A. M. Abd El-Aty

**Affiliations:** ^1^Institute of Quality Standard and Testing Technology for Agro-products, Shandong Academy of Agricultural Sciences, Jinan, China; ^2^Shandong Provincial Key Laboratory Test Technology on Food Quality and Safety, Jinan, China; ^3^College of Life Sciences and Food Engineering, Hebei University of Engineering, Handan, China; ^4^Key Laboratory of Molecular Medicine and Biotherapy, School of Life Sciences, Beijing Institute of Technology, Beijing, China; ^5^State Key Laboratory of Biobased Material and Green Papermaking, Qilu University of Technology, Shandong Academy of Sciences, Jinan, China; ^6^Department of Pharmacology, Faculty of Veterinary Medicine, Cairo University, Giza, Egypt; ^7^Department of Medical Pharmacology, Medical Faculty, Ataturk University, Erzurum, Turkey

**Keywords:** aptamer, fluorescent aptasensor, DNA intercalated dyes, lactoferrin detection, milk powder

## Abstract

Lactoferrin (Lf), an iron-binding glycoprotein, regulates the immune system. It has broad-spectrum antimicrobial activity and is critical for child physical growth and development. As a common additive in the dairy industry, it is crucial to quantify LF content. This study established a self-assembly and universal fluorescence aptasensor for detecting LF in milk powder based on structure-selective dyes of PicoGreen intercalated in the label-free aptamer. Herein, the aptamer functions as both a specific recognition element against targets and a fluorescent signal reporter integrated with structure-selective dyes. First, the aptamer folds into a three-dimensional spatial structure based on complementary base pairings and intermolecular weak non-covalent interactions. Then, the dye is intercalated into the minor groove structures of the aptamer and triggers its potential fluorescent property. When the target exists, the aptamer binds to it preferentially, and its space structure unfolds. This causes the freeing of the subsequent dye and decreases the corresponding fluorescence. Hence, the reflected fluorescence signals could directly determine the target concentrations. Under the optimum conditions, a good linear relationship (*R*^2^, 0.980) was obtained in the Lf range from 20 to 500 nM with a detection limit of 3 nM (2.4 mg/kg) and good specificity, as well as a reliable recovery of 95.8–105.1% in milk powder. In addition, the universality was also confirmed with a good performance by quickly changing the aptamers against other targets (chlorpyrifos, acetamiprid, bovine thyroglobulin, and human transferrin) or utilizing another fluorescence dye. Therefore, this self-assembly aptasensor provides a universal and concise strategy for effective detection.

## Introduction

Lactoferrin (LF) is an essential biofunctional iron-binding protein of the innate immune system with a molecular weight of approximately 80 kDa. It affects host defense and tumor growth inhibition and has antibacterial, antiviral, and antiparasitic activities (1, 2). Recently, LF has been applied in dairy products and commercial foods for non-breastfed infants as a functional component, promoting optimal growth (3, 4). In 2012, the GB14880-2012 “Food Nutrition Fortification Use Standard” issued by the National Health and Wellness Commission of the People’s Republic of China listed LF as a nutritional fortification and set the maximum use of LF at 100 mg/100 × g for infant foods. To date, different analytical techniques have been established (5). Although chromatographic methods, such as high-performance liquid chromatography (HPLC) (6, 7), high-performance capillary electrophoresis (HPCE) (8), and high-performance liquid chromatography-tandem mass spectrometry (HPLC-MS/MS) (9), have good reproducibility and high accuracy, they would require complex pretreatment processes and professional personnel to operate. In recent years, antibody-based biosensors, such as electrochemical sensors (10), surface-enhanced Raman spectroscopy (11), and enzyme-linked immunosorbent assays (12), have gradually been used to detect LF. However, the antibody is generally more expensive, complicated to prepare with animal immunization experiments, and requires strict conditions to ensure its stability. Hence, simple and convenient methods are of great significance for sensitive LF detection.

Nucleic acid aptamers are single-stranded deoxyribonucleic acid (DNA) or RNA sequences generated from a random oligonucleotide library through the systematic evolution of ligands by exponential enrichment (SELEX) (13, 14). They exhibit many advantages, such as small size, simple synthesis, good stability, and non-immunogenic nature, as viable alternatives to antibodies (15). Numerous aptasensors (16) have been recently established. Among them, fluorescence-based aptasensors are the most common type, featuring simplicity, fast response, high sensitivity, and universal applicability (17, 18). However, most of them require labeling aptamers or modifying bases with fluorescent groups, which are time-consuming and expensive, and the affinity of the aptamer to the target and the detection sensitivity are affected (19). Therefore, label-free fluorescent aptasensors have attracted much interest due to their flexible design and versatility.

As an essential type of single-stranded oligonucleotide, aptamers display three prominent characteristics for developing label-free fluorescent sensors (20, 21). (i) The aptamer exhibits multiple negatively charged phosphate functional groups, which result in the aggregation of positively charged small molecule probes and enable efficient probe fluorescence quenching, such as perylene (22), pyrene (23), silole (24), and tetraphenylethene (25) derivatives. (ii) Some special folded spatial conformations of aptamers, such as protoporphyrin (26), malachite green, thioflavin T (27), N-methyl mesoporphyrin IX (28), and DFHBI (29), could enable the intercalation of dyes to produce strong fluorescence (30). (iii) The aptamer can be easily hybridized with its complementary chain, forming a double helix conformation of double-stranded DNA (dsDNA) through hydrogen bonding from base pairing (31). Thus, some minor groove binding dyes could specifically intercalate into dsDNA, inducing exponential fluorescence enhancement and enabling sensitive detection of PicoGreen (PG) (32), SYBR Green I (SGI) (33), and AccuBlue (34). There are inherent base-complementary pairings in the self-assembly structure of the aptamers’ flexible folding. Therefore, the minor groove-binding dyes could also intercalate into the aptamer structure to fabricate various aptasensors.

Herein, a self-assembly and universal fluorescence aptasensor was designed for the sensitive detection of LF in milk powder based on the specific embedding dye PicoGreen (PG), of which there are few pieces of literature introducing PG-based self-assembly aptasensors. PG is a highly ultrasensitive fluorescent dye that does not fluoresce when it is free. Upon binding to dsDNA and inducing a >1,000-fold fluorescence enhancement (35), dsDNA detection concentrations as low as 25 pg⋅mL^–1^ were enabled. The aptamer against LF was screened using capillary electrophoresis (CE)-SELEX by our group in 2020 with good affinity (K_*D*_, 20.74 nM) and specificity (36). Without LF, the aptamers formed a specific spatial structure by self-assembly folding. The dyes recognized the minor groove structures and intercalated into them, generating a noteworthy augmentation of the fluorescence signal. In the presence of LF, the aptamer binds to it preferentially, and its space structure is unfolded. This caused the freeing of the subsequent dye and decreased the corresponding fluorescence. In addition, the universality was also confirmed against the other four targets or utilizing another dye. This label-free strategy achieved universal and sensitive detection only by “mix-and-detect” procedures, contenting the need to design simple and feasible aptasensors.

## Experimental

### Reagents and materials

Lactoferrin (LF, from bovine milk), α-lactalbumin (α-La), casein (CS), chlorpyrifos, β-lactoglobulin (β-Lg), serum albumin (SA), and acetamiprid were procured from Sigma-Aldrich (Shanghai, China). Bovine thyroglobulin (TG) and human transferrin (H-TF) were acquired from Shanghai Yuanye Bio-Technology Co., Ltd. (Shanghai, China). Pico Green (PG) was procured from Shanghai BioScience Co., Ltd. (Shanghai, China), and SYBR Green I (SGI) was secured from Thermo Fisher Scientific Inc. (Bartlesville, United States). Acetic acid was purchased from Sinopharm Chemical Reagent Co., Ltd. (Shanghai, China). Three different milk powders were obtained from a local store. The aptamers were synthesized by Sangon Biotechnology Co., Ltd. (Shanghai, China), and the aptamer sequences are listed in Supplementary Table 1.

A high-speed refrigerated centrifuge (Neofuge 15R, Heal Force, Shanghai, China) and a vortex coagulator (Vortex 2, IKA, Staufen, Germany) were used to process the milk powders. Ultrapure water with a specific resistance of 18.25 MΩ cm was obtained from a Millipore filtration system (Millipore, Bedford, MA, United States). The fluorescence intensity was scanned by a microplate reader (Synergy HTX, BioTek, Vermont, United States) with an excitation wavelength of 485 nm and an emission wavelength of 528 nm. The 50 μm id bare capillary with a total length of 32.6 cm (effective length 20.3 cm) was obtained from Sino Sumtech (Handan, Hebei, China). High-performance capillary electrophoresis (HPCE) equipped with a UV detector (214 nm) was supported by Hanon Group (Jinan, China).

### Preparation of the aptasensor

First, we optimized the concentration of PG. Then, 10 μL of PG at different concentrations (1 × , 3 × , 5 × , 7 × , 10 × , and 20 × ) was added to a solution containing 30 μL of 100 nM aptamer and incubated for 5 min at room temperature. The fluorescence intensities at 528 nm were recorded with a microplate reader, and the excitation wavelength was 485 nm.

LF aptamer (30 μL, 100 nM) was incubated with LF solution (60 μL, different concentrations) for 15 min at room temperature. Then, 10 μL of PG (5 × ) was added and reacted for 5 min, and the fluorescence values were determined by a microplate reader.

### Detection of lactoferrin in milk powder

A certain amount (0.04 g) of milk powder was accurately weighed and then dissolved in 30 mM acetic acid solution and centrifuged at 5,000 rpm for 15 min (to dissolve fat and sediment protein). The supernatant (approximately 400 μL) was diluted with an equal volume of ultrapure water and passed through a 0.22 μm filter membrane. First, we chose one commercially available milk powder labeled with no LF as a blank sample, and its matrix solution was spiked with standard LF at concentrations of 0, 10, 20, 50, 100, 200, 500, and 1,000 nM. The linear relationship between quenching efficiencies (F_0_–F)/F_0_ and LF concentrations was fitted.

Three LF concentrations of 50, 100, and 200 nM were chosen in the recovery assay. Before the blank milk powder treatment, the LF stock solutions were spiked into the milk powder. After that, the fluorescence intensities of the processed samples were recorded, and the recoveries were calculated according to the calibration curve constructed in the matrix. In the actual application, the milk powder with three different LF concentration contents was treated and measured, and the LF content was calculated from the fluorescence intensities.

### Universality of the aptasensor

Four targets were selected as follows: chlorpyrifos (350.59 g/mol), acetamiprid (222.68 g/mol), TG (660 kDa), and H-TF (76 kDa). Under the optimal experimental conditions, the applicability of each target was investigated. The flexibility is verified by utilizing another fluorescent dye, SGI. First, the optimal concentration of SGI in the sensor system was determined. Then, the detection performance of the sensing system using SGI as a fluorescent probe was investigated under optimal conditions.

### Capillary electrophoresis assay

The bare capillaries were rinsed with 1 M NaOH for 25 min and then with ultrapure water for 5 min. In the HPCE assay, sequential rinses for the capillary with 1 M NaOH, water, and running buffer solution each for 3 min were required between each run. The sample was injected at 0.5 psi for 20 s or the required time. The detection wavelength was set to 214 nm. During separation, a high voltage of 12 kV (the inlet as the anode) and a temperature of 22°C were maintained.

### The dsDNA aptasensor assay based on PicoGreen

A total of 20 μL of LF aptamer (100 nM) was incubated with 50 μL of different concentrations (300–3,000 nM) of LF standard solution for 15 min at room temperature. Then, 20 μL of 300 nM complementary DNA (cDNA) was added and incubated for 30 min. Subsequently, 10 μL of PG (7×) was added, and the fluorescence values were scanned with a microplate reader after 5 min.

## Results and discussion

### Principle and feasibility

**FIGURE 1:**
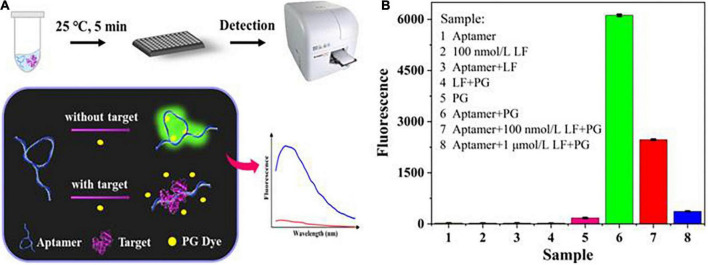
**(A)** The principle of the label-free aptasensor strategy for LF detection. **(B)** Feasibility verification of the aptasensor.

As depicted in Figure 1A, the sensing system for LF detection consisted of three parts: aptamers, PG dyes, and LF targets. Without LF, the aptamers formed a specific spatial structure by self-assembly folding due to the combined and additive effect of complementary base pairings and intermolecular weak non-covalent interactions (e.g., electrostatic and π-π interactions, hydrophobic effects, hydrogen bonding and van der Waals forces). At this stage, the PG dyes recognized the minor groove structures, intercalated into them, and stimulated their potential fluorescent properties, initiating a strong fluorescence. LF targets induced configuration changes in aptamers owing to their high-affinity bonding, concurrently forcing the amount of the inserted dyes to decrease and causing a significant reduction in the fluorescence signal. Hence, the LF target concentrations could be directly determined through the reflected fluorescence signals.

Figure 1B shows the feasibility of the sensing system (fluorescence spectra in Supplementary Figure 1A). The mixture of PG and aptamer generated a distinct fluorescence enhancement. In contrast, no single component produced substantially no fluorescence, which indicated that PG had availably intercalated into the partial spatial structures of the aptamer. Adding LF (100 nM, 1 μM) decreased the fluorescence, suggesting that the aptamer was more inclined to bind with its target LF, thus retaining more free PG dye. This phenomenon can also be explained by our previous molecular docking model (36) of aptamer/LF recognition that involved the 18 amino acids of LF and 17 key bases of aptamer, whose interaction would unfold the spatial structure of the aptamer and free the dyes. The result preliminarily proves that the experimental principle is feasible.

### Optimization of the aptasensor

The concentration and incubation time of PG were optimized to achieve optimal performance. Supplementary Figure 1B shows the effect of different concentrations of PG on the fluorescence signal. The maximum fluorescence intensity value was obtained when the PG concentration was increased to 5 × , indicating that at this concentration, PG can completely intercalate into the minor groove of the aptamer. Nevertheless, the fluorescence values tended to decrease when the concentration was more than 5 × because an excessively high PG concentration would cause a spatial site resistance effect and thus affect the fluorescence intensity. Therefore, 5 × PG was used for further experiments. In addition, the reaction time of PG also plays a vital role in the sensing process. Supplementary Figure 1C shows that the fluorescence intensity maintained a plateau within 5–30 min of incubation time. To improve the detection efficiency, the incubation time was determined to be 5 min.

### Sensitivity, specificity, and stability

**FIGURE 2:**
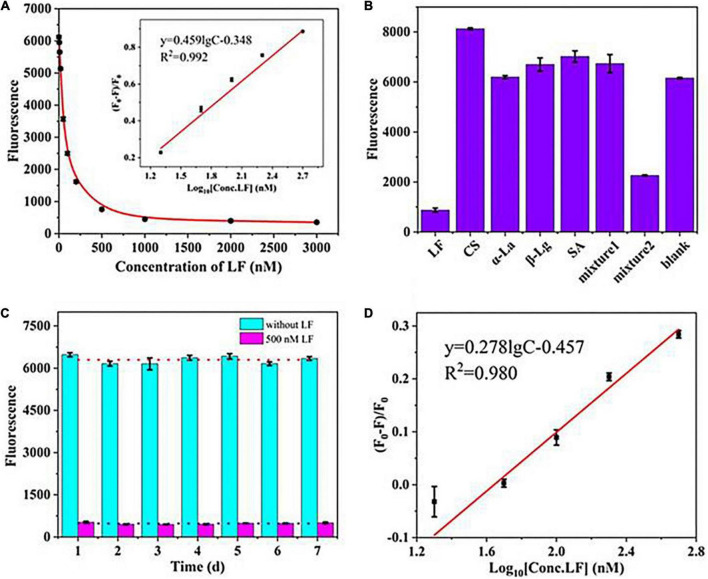
**(A)** The linear relationship between (F_0_–F)/F_0_ and the concentrations of LF. **(B)** The specificity of the aptasensor strategy. **(C)** Stability assessment of the fluorescence aptasensors. **(D)** The calibration curve in milk powder matrix.

The sensing performance was evaluated in terms of sensitivity, specificity, and stability. Figure 2A shows that the fluorescence intensity declined progressively as the concentration of LF increased from 0 to 3,000 nM and reached a plateau when the concentration surpassed 500 nM. The fluorescence quenching efficiency (F_0_-F)/F_0_ showed a good linear relationship with LF concentration in the range of 20–500 nM (in logarithmic form) with a correlation coefficient (*R*^2^) of 0.992 (Figure 2A inset image). The limit of detection (LOD, 3σ/k) was 2 nM. The value of (F_0_–F)/F_0_ is defined as the quenching efficiency, where F_0_ and F are the fluorescence values in the absence and presence of LF, respectively. The proposed aptasensor presented an available performance only through a simple “mix-and-detect” procedure.

To investigate the specificity of the sensing strategy, four types of high-abundance proteins commonly found in milk at high concentrations were tested, including serum albumin (SA), casein (CS), β-lactoglobulin (β-Lg), and α-lactalbumin (α-La). Figure 2B shows only LF, and mixture 2 (containing LF, CS, α-La, β-Lg, and SA) presented a noticeable reduction in fluorescence intensities in the sensing system. In contrast, the other proteins and mixture 1 (containing CS, α-La, β-Lg, and SA) led to almost no decrease even though their concentrations were 4-fold excess (2 μM) than the target LF. The results illustrated that the introduced self-assembled aptasensor presented good specificity for LF detection. However, mixture 2 of the control proteins (each at 2 μM) and LF (0.5 μM) still exhibited a slight influence on the fluorescence signal as LF (0.5 μM) only, which thereby suggested that the matrix effects of the sample need to be considered in practical applications.

To assess the stability, the fluorescence intensities of a blank sample and a standard LF of 500 nM were measured in an interday assay for seven consecutive days (Figure 2C). The coefficient of variation (CV) can relatively reflect the dispersion of experimental data and is also commonly used to evaluate the repeatability and stability of experiments (37). Higher CV values indicate higher detection errors, while lower values indicate more stable results. The calculated CV of the interday assay was 4.75%, wherein the low value demonstrated the good stability of the aptasensor.

### Lactoferrin determination in milk powder

The experiments were carried out in a milk powder matrix to assess the operability and usefulness of the introduced aptasensor in actual samples. One commercially available milk powder labeled with no LF was chosen as a blank sample, which was validated by a high-performance capillary electrophoresis (HPCE) assay (Supplementary Figure 2 an insert picture). After simple dissolution and centrifugation, the sample matrix was spiked with standard LF in the concentration range of 0–1,000 nM. Figure 2D shows that the fluorescence signal gradually decreased with increasing LF concentration. A good linear relationship was obtained over the range of 20–500 nM for the value of (F_0_-F)/F_0_ and the concentration of LF, and the LOD was calculated to be 3 nM (2.4 mg/kg). The detection limit meets China’s GB14880-2012, which shall not exceed 1.0 g/kg. Such high sensitivity could predict the potential LF content in milk powders. Although the slope presented a difference from that in the standard solution, the detection results of LF in matrixes could be quantitatively deduced by the calibration curve in the standard solution multiplied by an adjustment factor. In addition, the coefficient of determination (*R*^2^) in matrixes was greater than 0.98, showing good linearity in the analytical range. The results showed that the system fluorescence signal responded well to the LF concentration in the milk powder matrix. Furthermore, the aptasensor showed a good average recovery of 95.8–105.1%, with a relative standard deviation (RSD) of less than 4% in the milk powder sample (Table 1), which indicated that this developed aptasensor works well in practical applications.

**TABLE 1:** Recovery assay in milk powder using PG-based aptasensor (*n* = 3).

Added (nM)	Found (nM)	Recovery (%)	RSD (%)
50	47.9	95.8	3.6
100	98.2	98.2	3.9
200	210.1	105.1	1.4

### Universality verification

**FIGURE 3:**
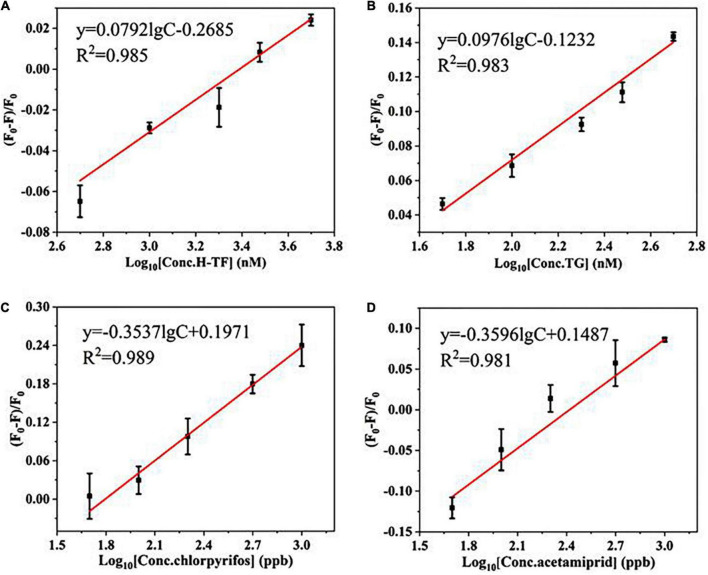
The linear relationship between (F_0_–F)/F_0_ and the concentrations of targets: **(A)** human transferrin (H-TF, 50–500 nM); **(B)** bovine thyroglobulin (TG, 50–500 nM); **(C)** chlorpyrifos (50–1,000 ppb); **(D)** acetamiprid (50–1,000 ppb).

In addition, the universality was confirmed by easily changing the aptamers against two other types of large molecular proteins (human transferrin and bovine thyroglobulin) and two kinds of small molecular pesticides (chlorpyrifos and acetamiprid). As shown in Figures 3A–D, the quenching efficiency (F_0_–F)/F_0_ values of all targets have good linear relationships with the target concentrations as well as good correlation coefficients (*R*^2^> 0.95). The results confirmed that the proposed strategy is likely to be a sensitive and universal aptasensor.

**FIGURE 4:**
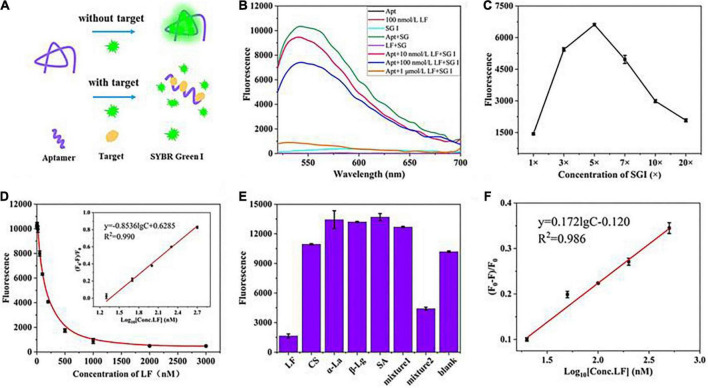
**(A)** The principle of the fluorescence aptasensor based on SGI. **(B)** Feasibility verification. **(C)** Optimization of SGI concentration. **(D)** Relationship between fluorescence intensity and the concentrations of LF. **(E)** Evaluation of aptasensor specificity. **(F)** The linear relationship was obtained in the milk powder matrix between (F_0_–F)/F_0_ and the concentrations of LF.

Furthermore, the flexibility of the fluorescent aptasensor was also verified utilizing another fluorescent dye, SYBR Green I (SGI) (Figures 4A–F), with similar characteristics to PG, enabling dsDNA detection concentrations as low as 20 pg/mL (38). In the range of 20–500 nM, good linear relationships were obtained in the standard solution (*R*^2^, 0.990) and the milk powder matrix (*R*^2^, 0.986) with LODs of 5.4 and 5.9 nM, respectively. Meanwhile, the aptasensor exhibited good selective specificity and spiked recovery (106.2–108.5%, Supplementary Table 2), indicating that the strategy is feasible in practical applications.

Furthermore, we applied this strategy to determine the LF content in three milk powders, which were also quantified using the HPCE method (8) (Supplementary Figure 2). The content of LF in milk powders detected by the two methods is listed in Supplementary Table 3, and the results show that the HPCE method is roughly consistent with our aptasensor. However, the aptasensor took only 20 min to complete a quick analysis in all samples, which was much shorter than that required by the commercial CE of approximately 120 min for all samples.

### Comparison with other methods

The performance comparison of this aptasensor with other LF methods is shown in Table 2. Radial immunodiffusion is one of the simplest methods; however, it has poor sensitivity. HPCE, HPLC, and SPR techniques have shown good performance, but they are expensive and require professional technicians. Microfluidic paper and ELISA have high accuracy, but both are based on the immunoreaction between LF and its antibody, which are expensive reagents and complicated to prepare. Among the detection methods, the present work reported a very low LOD of 0.16 μg/mL (2 nM). Although Huang et al. (10) reported a higher sensitivity with a lower LOD, electrochemical sensors still rely heavily on expensive electrochemical systems with high resolution, and cyclic voltammetry requires 20 scans to obtain reliable results. The self-assembled fluorescent aptasensor presented a low dependence on instruments and an available performance with a simple “mix-and-detect” procedure, high sensitivity and time savings compared with other reported methods for LF detection.

**TABLE 2:** Performance comparison of various methods for detecting LF.

Method	Linear range	LOD	References
Radial immunodiffusion	250–4,000 μg/mL	–	(39)
Capillary electrophoresis	10–500 μg/mL	5.0 μg/mL	(40)
Liquid chromatography	10–1,000 μg/mL	500 μg/mL (liquid samples)	(41)
		420 μg/mL (solid samples)	
Microfluidic paper	0–1,000 μg/mL	110 μg/mL	(42)
ELISA	5–600 × 10^–3^ μg/mL	3.23 × 10^–3^ μg/mL	(43)
Surface plasmon resonance	0–1 μg/mL	1.11 × 10^–3^ μg/mL	(44)
Electrochemical sensor	10^–5^–1 μg/mL	4.9 × 10^–6^ μg/mL	(10)
Self-assembly fluorescent aptasensor	20–500 nM (1.6–40 μg/mL)	2 nM (0.16 μg/mL)	This work

## Conclusion

In conclusion, we presented a label-free and universal aptasensor for LF detection in milk powder based on the dual functions of aptamers as a specific recognition element against targets and a fluorescent signal reporter integrated with structure-selective dyes. The direct-recognition aptasensor avoids the synthesis, consumption and complex optimization of the complimentary chains generally required in dsDNA-based aptasensors and gains a better performance (LOD, 2 nM vs. 205 nM, Supplementary Figure 3) by a facile “mix-and-detect” operation. Furthermore, this strategy has been demonstrated for four target detection methods (TG, H-TF, chlorpyrifos, and acetamiprid) and two fluorescence dyes (PG and SGI). Therefore, the aptasensor provides expansive prospects for developing a simple, general, cost-effective detection.

## Data availability statement

The original contributions presented in this study are included in the article/Supplementary material, further inquiries can be directed to the corresponding author/s.

## Author contributions

CZ: writing original draft, data curation, funding acquisition, and project administration. JL: investigation, validation, data curation, and formal analysis. LL: data curation and formal analysis. HQ and TL: formal analysis. MY: funding acquisition. FQ: writing – review and editing, and funding acquisition. AA: validation, formal analysis, and writing – review and editing. All authors contributed to the article and approved the submitted version.
