# Supplementary material for: Self-assembly and label-free fluorescent aptasensor based on deoxyribonucleic acid intercalated dyes for detecting lactoferrin in milk powder
Source: Front Nutr. 2022 Sep 15;9:992188. doi: 10.3389/fnut.2022.992188 (PMC9521185; doi:10.3389/fnut.2022.992188)
Supplement: Supplementary file 1 [file Data_Sheet_1.docx]

**Electronic Supplementary Material**

**Self-assembly and label-free fluorescent aptasensor based on DNA intercalated dyes for detecting lactoferrin in milk powder**

Jiahui Liu^a, b, c^, Tengfei Li^c^, Hongwei Qin^a, b^, Linsen Li^d^, Mengmeng Yan^a, b^*, Chao Zhu^a, b^*, Feng Qu^d^, A. M. Abd El-Aty^e^

*^a^Institute of Quality Standard and Testing Technology for Agro-products, Shandong Academy of Agricultural Sciences, Jinan 250100, China*

*^b^Shandong Provincial Key Laboratory Test Technology on Food Quality and Safety, Jinan, 250100, China*

*^c^Life Sciences and Food Engineering, Hebei University of Engineering, Handan 056000, China*

*^d^Key Laboratory of Molecular Medicine and Biotherapy, School of Life Science, Beijing Institute of Technology, Beijing 100081, China*

*^e^Department of Pharmacology, Faculty of Veterinary Medicine, Cairo University, 12211-Giza, Egypt*

*Corresponding authors. Email addresses: [ndytzhuchao@126.com](mailto:ndytzhuchao@126.com); [ynky202@163.com](mailto:ynky202@163.com)

**Table S1.** The aptamer sequences were employed in this study.

| Aptamer | Sequences (from 5’ to 3’) |
| --- | --- |
| LF | TGGTGCTGCCCCTAGTCTCCGGCTGATAGCTGCTTCTTGG |
| TG | CCTAACCGATATCACACTCACCGCGTGAGCGGGGAGGCGATGCCCAGGCTAACTTGACTCAGTTGGTCGTCATTGGAGTATC |
| H-TF | AGCAGCACAGAGGTCAGATGCGACTTGCCTATCGGCATGACACAATCTTTTGGAGCGTAACCTATGCGTGCTACCGTGAA |
| chiorpyrifos | CCTGCCACGCTCCGCAAGCTTAGGGTTACGCCTGCAGCGATTCTTGATCGCGCTGCTGGTAATCCTTCTTTAAGCTTGGCACCCGCATCGT |
| acetamiprid | CTGACACCATATTATGAAGA |

| 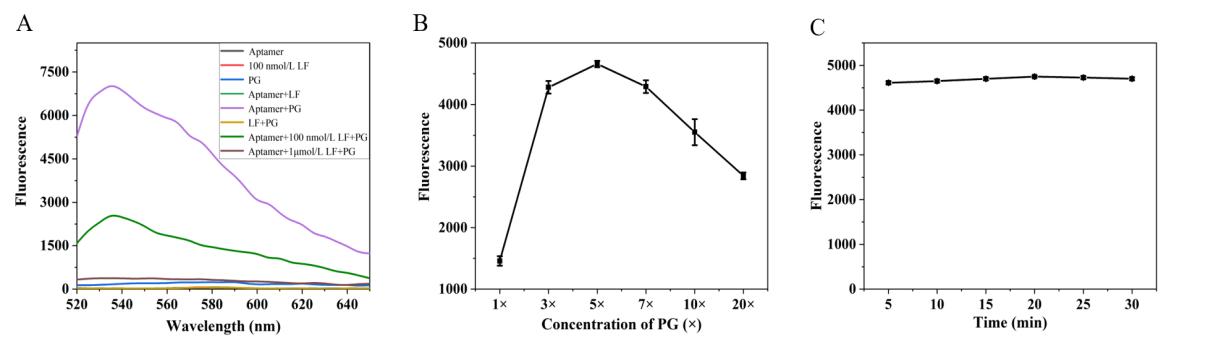 |
| --- |
| **Fig. S1** (A) Feasibility verification. (B) The effect of different concentrations of PG. (C) Optimization of PG incubation time. |

**Table S2** Recovery assay in milk powder by using SGI-based aptasensor (n=3).

| Added (nM) | Found (nM) | Recovery (%) | RSD (%) |
| --- | --- | --- | --- |
| 50 | 53.7 | 107.4 | 4.9 |
| 100 | 108.5 | 108.5 | 4.1 |
| 200 | 212.4 | 106.2 | 2.1 |

| \| HPCE method was employed to verify the accuracy of this method. LF was quantified by the HPCE method (Fig. S2), and the contents of LF in the milk powder detected by both methods are listed in Table S3.  **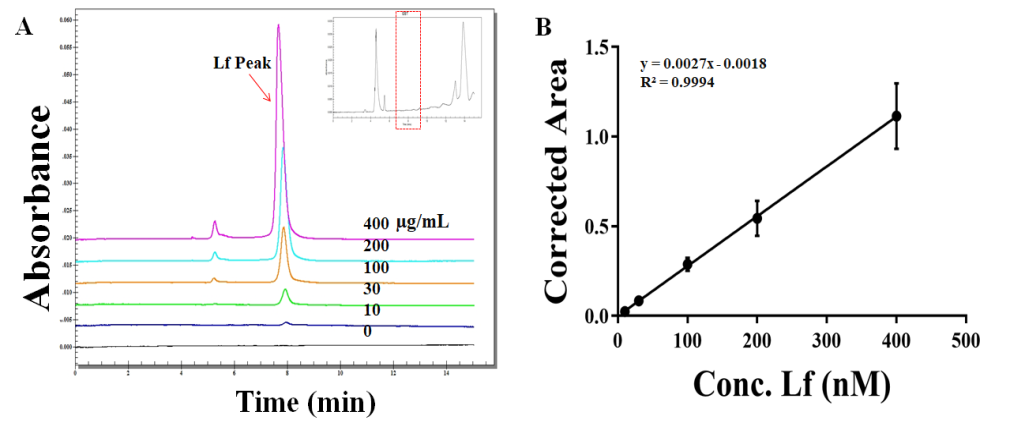** \| \| --- \| |
| --- | --- |
| **Fig. S2.** CE validation assay. |

**Table S3.** Detection of LF in milk powder samples.

| Samples | Measured value (mg/100 g) | |
| --- | --- | --- |
|  | This work | HPCE |
| 1 | 28.00 | 22.27 |
| 2 | 10.75 | 14.58 |
| 3 | 0 | 0 |

**The dsDNA aptasensor method based on PG**

The principle of the PG dye-based dsDNA aptasensor is shown in Fig. S3A. The aptamer forms a double-stranded structure with its complementary strand, and PG specifically binds to the dsDNA and generates high fluorescence intensity; however, when the target is present, the aptamer binds its target preferentially, resulting in a reduction in dsDNA formation bound to PG and a significant decrease in fluorescence intensity. The optimal experimental conditions were obtained by optimizing the concentrations of PG and complementary strands (Fig. S3B, C). The fluorescence intensity of the LF concentration in the range of 0.3-3 μM was measured under the optimized experimental conditions, and the inset in Fig. S3D shows a good linear relationship (*R^2^*=0.994) between LF in the concentration range of 0.3-1.5 μM and fluorescence quenching efficiency (F_0_-F)/F_0_, and the calculated LOD was 205 nM.

| 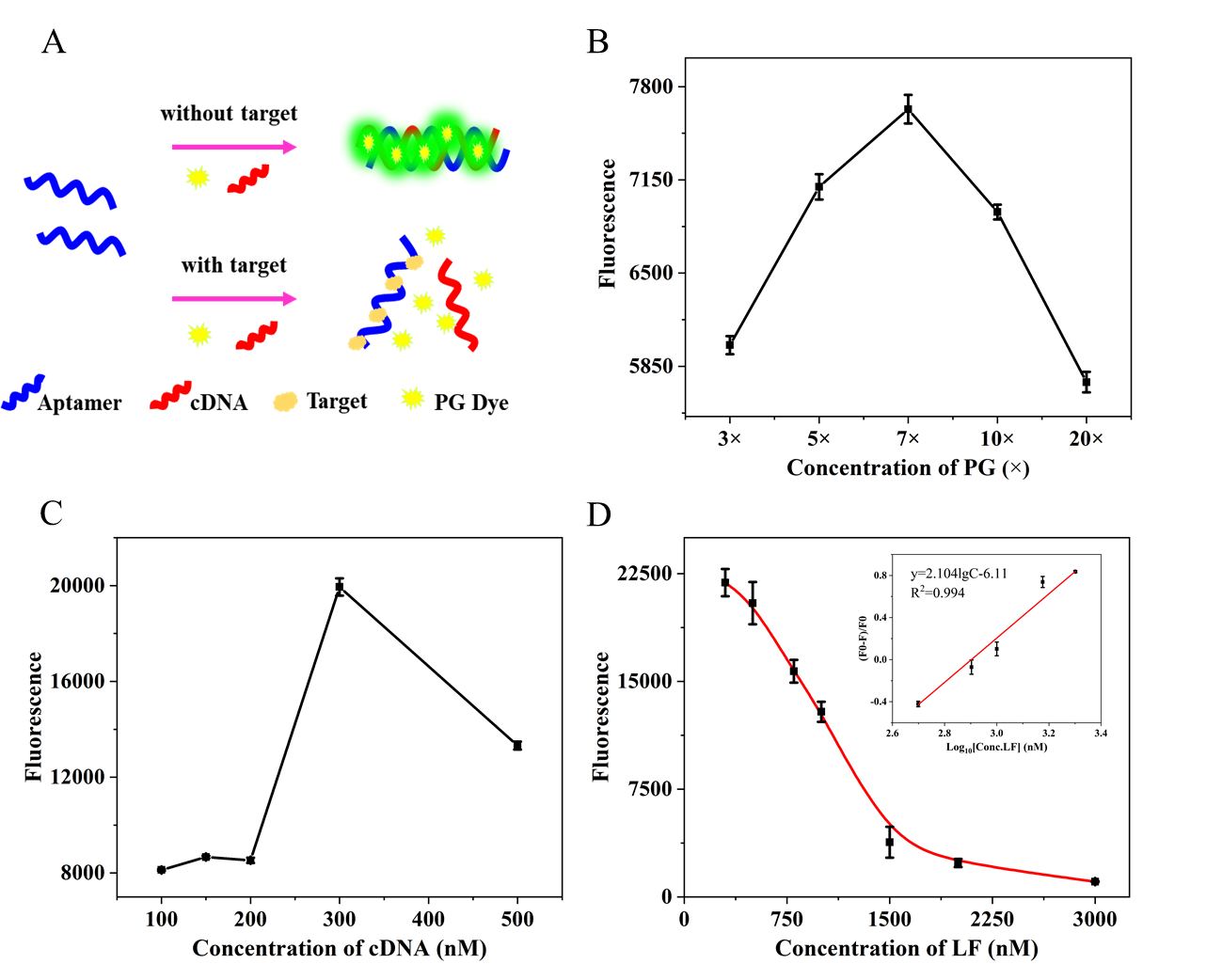 |
| --- |
| **Fig. S3** (A) The principle of the dsDNA-aptasensor method based on PG. (B) Optimization of PG concentration. (C) Optimization of cDNA concentration. (D) Linear relationship between fluorescence intensity and the concentrations of LF. |
